# Synthesis of compositionally controllable Cu_2_(Sn_1−x_Ge_x_)S_3_ nanocrystals with tunable band gaps

**DOI:** 10.1007/s11051-016-3439-5

**Published:** 2016-06-15

**Authors:** Qingshuang Liang

**Affiliations:** State Key Laboratory of Inorganic Synthesis and Preparative Chemistry, College of Chemistry, Jilin University, Changchun, 130012 People’s Republic of China

**Keywords:** Nanocrystals, Compositionally controlled, Tunable band gap, Ge inclusion, Solar energy conversion

## Abstract

In this work, we show that compositionally controlled Cu_2_(Sn_1–x_Ge_x_)S_3_ nanocrystals can be successfully synthesized by the hot-injection method through careful tuning the Ge/(Sn+Ge) precursor ratio. The band gaps of the resultant nanocrystals are demonstrated to be linearly tuned from 1.45 to 2.33 eV by adjusting the composition parameter x of the Ge/(Sn+Ge) ratio from 0.0 to 1.0. The crystalline structures of the resultant NCs have been studied by the X-ray diffraction (XRD), high-resolution transmission electron microscopy (HRTEM), select area electron diffraction (SAED), and Raman spectroscopy. A ligand exchange procedure is further performed to replace the native ligands on the surface of the NCs with sulfur ions. The photoresponsive behavior indicates the potential use of as-prepared Cu_2_(Sn_1–x_Ge_x_)S_3_ nanocrystals in solar energy conversion systems. The synthesis of compositionally controlled Cu_2_(Sn_1–x_Ge_x_)S_3_ nanocrystals reported herein provides a way for probing the effect of Ge inclusion in the Cu-Sn-S system thin films.

**Electronic supplementary material:**

The online version of this article (doi:10.1007/s11051-016-3439-5) contains supplementary material, which is available to authorized users.

## Introduction

Low-cost thin-film solar cell light-harvesting materials have drawn lots of attention in recent years (Ramasamy 2012; Fan et al. 2014). In particularly, quaternary semiconductors Cu_2_ZnSnS_4_ (CZTS) and Cu_2_ZnSnSe_4_ (CZTSe), in benefit of their earth-abundant constituents, optimal band gap, and high absorption coefficient, have attracted a great interest as promising substitutes of In- and Ga-free absorber materials for photovoltaic applications (Guo et al. 2010; Riha et al. 2011; Liang Shi et al. 2011; Carrete et al. 2013; Zhou et al. 2013; Zou et al. 2013; Siebentritt and Schorr 2012). Yet due to the increased number of component elements in the materials, the synthesis of these materials is relatively more difficult than those of binary and ternary semiconductors (Chen et al. 2013). Recently, with fewer elements and perhaps reduced complexity, p-type ternary Cu-Sn-S system semiconductors have also been considered as candidates for low-cost thin-film solar cell absorbers, and an increasing number of studies have been reported (Zhai et al. 2011; Berg et al. 2012; Norako et al. 2012; Ahmadi et al. 2013; Nomura et al. 2013; Yang et al. 2013). Kuku and Fakolujo first reported a power conversion efficiency of 0.11 % based on a Schottky-type Cu_2_SnS_3_/In solar cell (Kuku and Fakolujo 1987). And then Koike et al. fabricated Mo/Cu_2_SnS_3_/CdS/ZnO: Al thin-film solar cells and demonstrated a solar energy conversion efficiency of 2.84 % (Koike et al. 2012). Compared to other thin-film solar cell light-harvesting materials, however, the power conversion efficiencies of Cu_2_SnS_3_-based solar cell devices are still relatively low. Optimizing the band gap and improving the performances of the Cu_2_SnS_3_-based solar cell devices is urgently needed and should be of great significance.

The optical band gap plays a significant role to determine the optical and electrical properties of photovoltaic (PV) applications. It is well known that the indium-based CuInSe_2_ device performance achieves solar cell efficiency maximum by substituting In with Ga and optimizing the In:Ga ratio (Jackson et al. 2011; Dilena et al. 2013; Jiang Tang et al. 2008). Substitution of In with the lighter group III element, Ga, widens the band gap. Similarly, band gap tuning and improvements to CZTS devices have also been reported by substitution of Sn with the lower atomic number and isoelectronic Ge (Ford et al. 2011; Bag et al. 2012). First principle calculations have indicated that the smaller Ge atoms strengthen the s–s and s–p level repulsion between the Ge and S atoms, leading to an increase in the anti-bonding conduction band minimum (Chen et al. 2010). Widening the band gap and improving the device performance by substituting Sn with Ge should be also applied to the Cu-Sn-S system. Motohiro et al. recently have reported the incorporation of Ge to the Cu_2_SnS_3_ film solar cell, and a significant improvement of the power conversion efficiency appeared (Umehara et al. 2013). Solar cell fabricated from Cu_2_SnS_3_ film yielded a power conversion efficiency of 2.13 %, while the Cu_2_Sn_1–x_Ge_x_S_3_ film with a Ge/(Sn+Ge) ratio 0.17 yielded devices with an efficiency of 6 %. It is inspiring that the performances of the Cu_2_SnS_3_-based solar cell devices could indeed be improved by the incorporation of Ge, and it is also surprising that the inclusion of such little amount (0.17) of Ge could induce so large device efficiency increase. Unfortunately, in Motohiros’ work, only one component of Ge-doping was reported, and the effect of different amount of Ge inclusion to the Cu_2_SnS_3_-based devices has not been investigated.

Here, in this work, we show that compositionally controlled Cu_2_(Sn_1–x_Ge_x_)S_3_ nanocrystals, in which the contents of Sn and Ge can be varied across the entire range (0 ≤ *x* ≤ 1), have been synthesized via a facile solution-based route. The band gaps of the resultant nanocrystals are demonstrated to be linearly tuned from 1.45 to 2.33 eV by adjusting the composition parameter *x* of the Ge/(Sn+Ge) ratio from 0.0 to 1.0. The crystalline structures of the resultant NCs have been studied by the X-ray diffraction (XRD), high-resolution transmission electron microscopy (HRTEM), select area electron diffraction (SAED), and Raman spectroscopy. A ligand exchange procedure is further performed to replace the native ligands on the surface of the NCs with sulfur ions. The photoresponsive behavior indicates the potential application of as-prepared Cu_2_(Sn_1−x_Ge_x_)S_3_ nanocrystals in solar energy conversion systems. The synthesis of compositionally controlled Cu_2_(Sn_1−x_Ge_x_)S_3_ nanocrystals reported herein provides a way for probing the effect of Ge inclusion in the Cu-Sn-S system thin films.

## Experimental section

### Chemicals

All chemicals were used as received without further purification. Tin (II) bromide (SnBr_2_, 99 %) and Germanium (IV) chloride (GeCl_4_, 99.9999 %) were obtained from Sigma-Aldrich. Sodium sulfide nonahydrate (Na_2_S·9H_2_O, 98 %) was purchased from Alfa Aesar. Cupric acetate (Cu (OAc) _2_, 99 %), sulfur powder (99.99 %), 1-dodecanethiol (98 %), and oleylamine (80–90 %) were obtained from Aladdin. Hexane (analytical reagent), ethanol (analytical reagent), formamide (analytical reagent), and acetone (analytical reagent) were purchased from Beijing chemical works.

### Synthesis of Cu_2_(Sn_1-x_Ge_x_)S_3_ nanocrystals

The synthesis of Cu_2_(Sn_1−x_Ge_x_)S_3_ nanocrystals was carried out in oleylamine (OAm) solution by a hot-injection method. In a typical synthesis, 1 mmol Cu (OAc) _2_ (0.1816 g), 0.25 mmol SnBr_2_ (0.0.0696 g), and 10 mL OAm were loaded to a 100 mL three-neck flask attached with a Schlenk line. The flask was degassed by a vacuum pump for 30 min to remove water and other low-boiling point impurities at 125 °C. Then, 0.25 mmol GeCl_4_ (0.25 mL of 1 M solution of GeCl_4_ in air-free oleylamine) was injected into this solution. The temperature was subsequently increased to 160 °C, followed by swift injection of 1.5 mmol (0.048 g) sulfur dissolved in 2 mL 1-dodecanethiol (DDT). The reaction solution was then heated to 280 °C in 10 min and maintained at this temperature for 3 h with continuous vigorous stirring. After the mixture was cooled to room temperature, 5 mL of hexane and 25 mL of ethanol were added, and the mixture was sonicated for 5 min to remove all the free ligands and the unreacted precursors. The solution was centrifuged at 9000 rpm for 10 min. The upper layer liquid was decanted, and the isolated solid was dispersed in hexane and reprecipitated by adding ethanol. The centrifugation and precipitation procedure was repeated three times, and the final products were redispersed in hexane or dried under vacuum for further measurements.

### Ligand exchange procedures

The ligand exchange process was performed according to the literatures with some modifications (Nag et al. 2011; Stolle et al. 2012). 0.863 g of Na_2_S·9H_2_O was dissolved in 20 mL formamide (FA). For a typical ligand exchange, 50 mg of the as-prepared Cu_2_Sn_0.5_Ge_0.5_S_3_ NCs were dispersed into 5 mL of hexane and then mixed with 5 mL of the FA solution. The mixture was stirred for about 2 h, leading to a complete phase transfer of Cu_2_Sn_0.5_Ge_0.5_S_3_ NCs from hexane to FA phase. The phase transfer could be easily monitored by the color change of hexane (black to colorless) and FA (colorless to black) phase. The residual organics were removed by adding hexane and decanting the mixture three times. The nanocrystals were precipitated by adding 5 mL of acetone and centrifuging at 9000 rpm for 1 min.

### Devices fabrication

Thin films for optoelectronic properties characterizations were deposited by spin casting nanocrystals dispersed in toluene. The fabrication details of the device structure are as follows: an patterned indium tin oxide (ITO)-coated glass was used as the substrate and cleaned by successive ultrasonic treatment in acetone and isopropyl alcohol and then dried at 120 °C for 30 min. The ITO glass was then subjected to UV-Ozone treatment for 10 min. A 30-nm-thick Poly (ethylenedioxythiophene): poly (styrenesulfonate) (PEDOT: PSS, Baytron P4083) layer was spin coated onto the ITO glass and baked at 120 °C for 30 min in ambient. Subsequently, the Cu_2_Sn_0.5_Ge_0.5_S_3_ film was fabricated by spin casting the concentrated nanocrystals toluene dispersion on the substrate. Then a 5-nm-thick PF-EP buffer layer was spin-coated onto the Cu_2_Sn_0.5_Ge_0.5_S_3_ layer from ethanol solution. The substrates were then transferred into an evaporator and pumped down to 4 × 10^−4^ Pa to deposit 100-nm-thick aluminum (Al) cathodes. Post-thermal annealing was carried out at 140 °C for 3 min on a hot plate inside a nitrogen-filled glove box and the devices were encapsulated for measurement.

### Characterization

UV–visible absorption spectra were recorded on a Cary 50 Scan UV–visible spectrophotometer (Varian, USA). X-ray power diffraction (XRD) patterns were recorded on a D8 Focus diffractometer (Bruker) with a Cu K_α1_ radiation source (*λ* = 0.15406 nm). The compositions of the as-prepared Cu_2_(Sn_1−x_Ge_x_)S_3_ nanocrystals were determined quasi-quantitatively by a Hitachi S-4800 high-resolution FE-SEM equipped with a Bruker AXS XFlash detector 4010, at an acceleration voltage of 20 kV. Transmission electron microscopy (TEM) and high-resolution (HR) TEM were performed on a FEI Tecnai G2 F20 with an accelerating voltage of 200 kV. Raman spectra were recorded on a Renishaw inVia micro-Raman spectrometer. Infrared spectra were collected on a VERTEX Fourier transform infrared (FTIR) spectrometer (Bruker). Thermogravimetric analysis (TGA) was carried out using a STA 449 F3 simultaneous thermal analyzer. Current density–voltage (*J*-*V*) characteristics of the PV cells were measured using a computer controlled Keithley 236 source meter under AM1.5G illumination from a calibrated solar simulator with irradiation intensity of 100 mW/cm^2^.

## Results and discussion

The composition of the resulting Cu_2_(Sn_1−x_Ge_x_)S_3_ quaternary nanocrystals were determined by energy-dispersive X-ray spectroscopy (EDS), and the results are listed in Table 1. Increasing the ratios of Ge/(Sn+Ge) in the precursors results in an increase of the Ge contents in the final products, and the Cu: Ge: Sn: S mole ratios in the products were in close agreement with the ratios of the precursors, demonstrating successful formation of composition tunable-alloyed Cu_2_(Sn_1−x_Ge_x_)S_3_ NCs with the variation of Ge/(Sn+Ge) in the entire compositional range (0 ≤ *x* ≤ 1).

**Table 1:** Composition analysis and band gap energies of Cu_2_(Ge_x_Sn_1−x_)S_3_ nanocrystals

Target compound	Cu:Ge:Sn:S precusor ratio	Cu:Ge:Sn:S ratio measured by EDS	*E*_g_ (eV)
Cu_2_SnS_3_ (*x* = 0)	2:0:1:3	1.85:0.00:1.00:2.98	1.46 (2)
Cu_2_(Ge_0.26_Sn_0.74_)S_3_(*x* = 0.26)	2:0.3:0.7:3	2.15:0.26:0.74:2.96	1.71 (2)
Cu_2_(Ge_0.5_Sn_0.5_)S_3_ (*x* = 0.5)	2:0.5:0.5:3	1.93:0.50:0.50:3.04	1.86 (1)
Cu_2_(Ge_0.74_Sn_0.26_)S_3_ (*x* = 0.74)	2:0.75:0.25:3	2.09:0.74:0.26:2.95	2.02 (1)
Cu_2_GeS_3_ (*x* = 1)	2:1:0:3	2.03:1.00:0.00:2.90	2.33 (3)

**Fig. 1:**
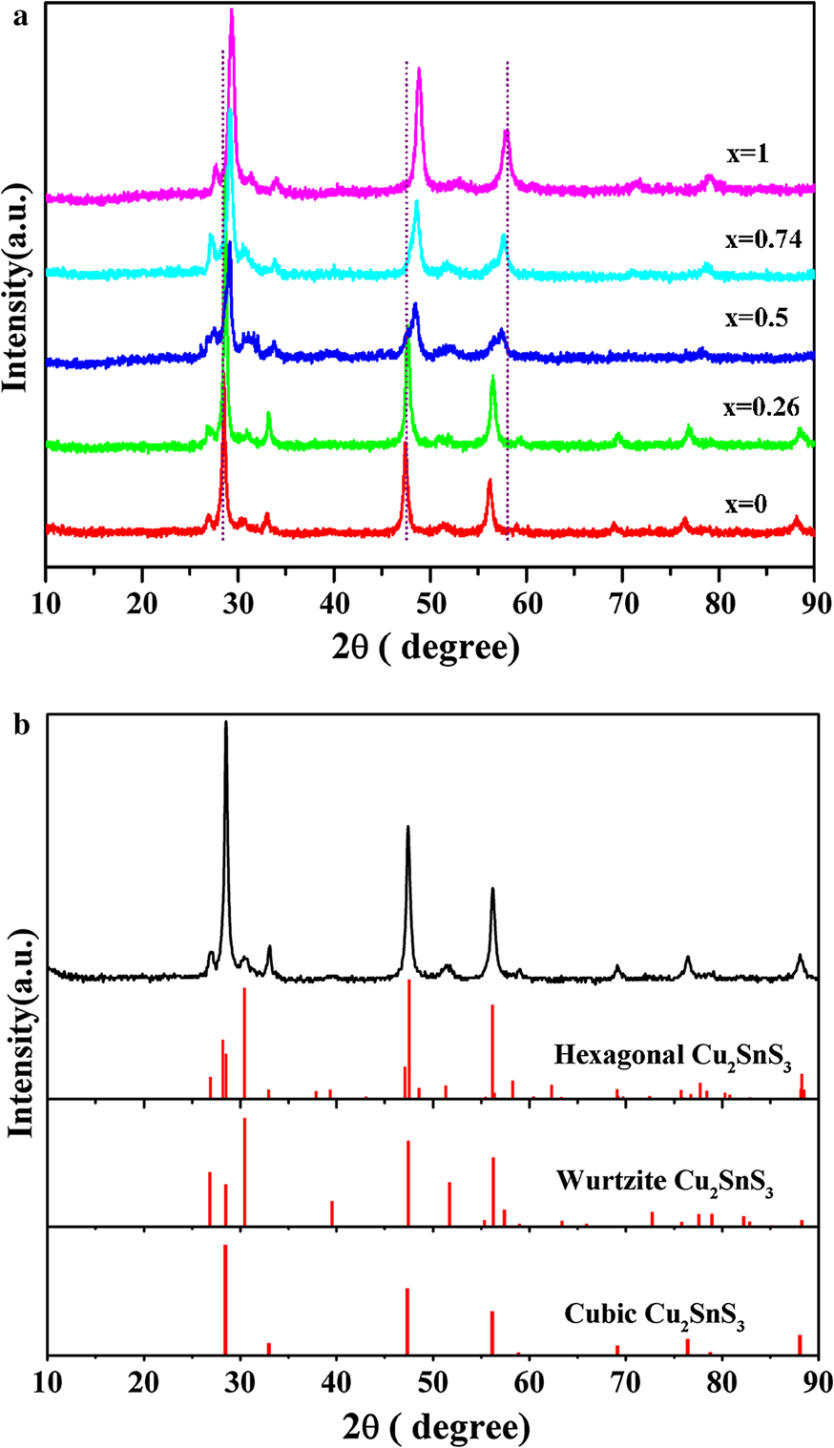
**a** XRD patterns of Cu_2_(Sn_1−x_Ge_x_)S_3_ nanocrystals for various values of x. **b** XRD pattern of the as-prepared Cu_2_SnS_3_ NCs and simulated XRD pattern of hexagonal, wurtzite, and cubic structures

**Fig. 2:**
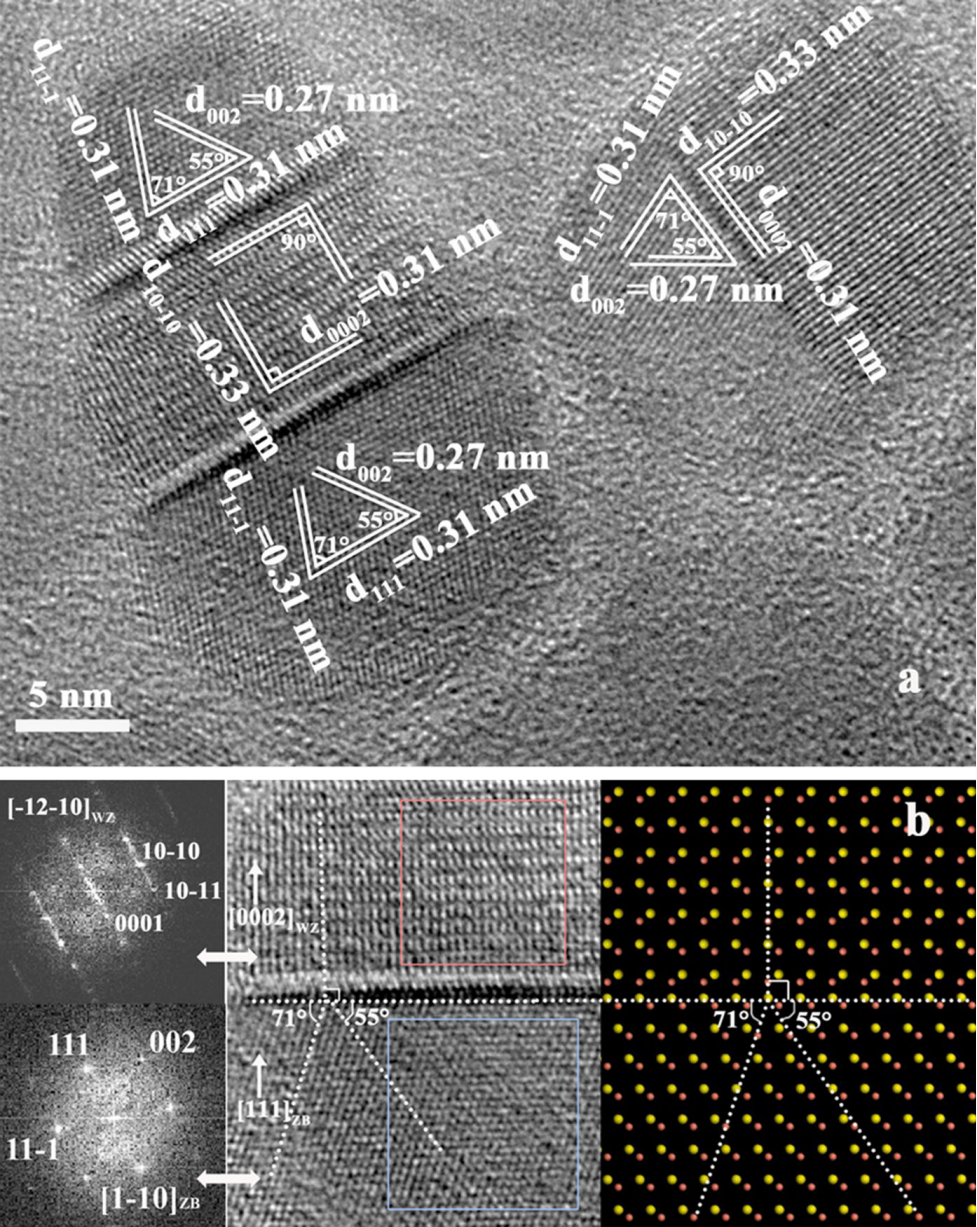
**a** HRTEM image of the as-prepared Cu_2_SnS_3_ NCs with typical staking faults. **b** FFT images of different area in Cu_2_SnS_3_ NCs HRTEM image and the crystal model of the interface of ZB-and WZ-derived structures (the *orange* and *dark red balls* represent cation and anion atoms, respectively). (Color figure online)

Figure 1(a) shows the powder XRD patterns of a series of Cu_2_(Sn_1–x_Ge_x_)S_3_ NCs synthesized with different Ge/(Sn+Ge) precursor ratios of 0.0, 0.3, 0.7, and 1.0, respectively. The diffraction peaks show a systematic shift to higher angle with increasing Ge contents, which may have ruled out the separated nucleation of Cu_2_SnS_3_ and Cu_2_GeS_3_ during the growth of the Cu_2_(Sn_1–x_Ge_x_)S_3_ NCs. The shift in the diffraction peaks is attributed to the reducing lattice constants due to the substitution of significantly larger Sn^4+^ with the smaller Ge^4+^ in the Cu_2_(Sn_1–x_Ge_x_)S_3_ lattices. It was found that the XRD patterns cannot be indexed to any existing patterns in the standard JCPDS database, not even those of the Cu_2_S, SnS_2_, and GeS_2_, or their mixtures. Similar XRD patterns of Cu-Sn-S system nanocrystals have been reported in the literatures (Wu et al. 2007; Wang et al. 2013; Maria Ibáñez et al. 2012). Wu et al. had observed that such XRD pattern was resembled with the XRD pattern of the hexagonal-CuSe pattern and assigned the crystalline structure of the relevant Cu_2_SnS_3_ nanocrystals to the hexagonal phase (Wu et al. 2007). Whereas, in the report of the synthesis of Cu_2_SnSe_3_ tetrapod nanocrystals with a cubic core and four wurtzite arms by Wang’s group, the XRD pattern of the CTSe tetrapods also displayed the similar XRD pattern (Wang et al. 2013). To acquire the accurate crystalline structure of the as-prepared Cu_2_(Sn_1−x_Ge_x_)S_3_ NCs, a close investigation of XRD, high-resolution transmission electron microscopy (HRTEM), select area electron diffraction (SAED), and Raman spectroscopy on the multicomponent Cu_2_(Sn_1−x_Ge_x_)S_3_ nanocrystals was performed. As shown in Fig. 1b, the experimental pattern of the as-prepared Cu_2_SnS_3_ NCs matches well with the simulated one of hexagonal structure with *a* = 3.836 Å, and *c* = 18.82 Å. Also, it is reasonable to consider that the as-prepared Cu_2_SnS_3_ NCs were component with the cubic zinc blende (ZB) and hexagonal wurtzite (WZ) mix-phase. The broadening peaks due to the nanostructure further obscure proper identification. Therefore, it is difficult to distinguish whether the as-prepared Cu_2_SnS_3_ NCs were component with the cubic and wurtzite mix-phase or only the pure hexagonal structure from the XRD. Since the wurtzite structure also belongs to hexagonal crystal system, based on lattice spacing and geometry of FFT in the HRTEM characterization also can not solve the puzzle. Carefully analyzed the HRTEM images, however, we noticed that multiple stacking faults existed in many as-prepared nanocrystals. HRTEM image with typical stacking faults and their corresponding fast Fourier transform (FFT) images of different area are showed in Fig. 2a, b. Stacking faults-induced phase transformation had been reported in similar semicondutors (Maria Ibáñez et al. 2012; Fan et al. 2012). Yu et al. had reported the epitaxial growth of zinc blende phase on wurtzite CZTSSe nanoparticles (Fan et al. 2012). Stacking faults at the interface of those two structures were observed. In the synthesis of polytypic Cu_2_GeSe_3_ nanoparticles with disordered cation position, Cabot et al. had found that periodicity of the twins change the atomic plane stacking, inducing the intermix of WZ structure into the ZB phase (Maria Ibáñez et al. 2012). They also found that when the reaction temperature is in the range of 273–300 °C, multiphase WZ–ZB structure Cu_2_GeSe_3_ nanoparticles were produced. While in our system, the reaction temperature is 280 °C. The ZB-derived and the corresponding WZ-derived structures differ in their atom stacking sequence along [111]_ZB_ and [0001]_WZ_ directions. The stacking sequence of ZB-derived structure is abcabc while for WZ-derived structure it is abab. The presence of a stacking fault in the cubic zinc blende or hexagonal wurtzite phase can periodically change the atomic plane stacking, forming a mix-phase structure. The lattice parameters calculated from selected area electron diffraction (SAED) patterns (Figure S 1) of several randomly chosen regions of the Cu_2_SnS_3_ nanocrystals both match well with the values calculated from the XRD pattern for WZ-ZB structure Cu_2_SnS_3_. At the Raman spectrum (Figure S 2), the peaks observed at 301, 354 cm^−1^ confirm the presented cubic (*F*-43 m) structure Cu_2_SnS_3_ in the as-prepared Cu_2_SnS_3_ NCs. Based on the above discussions, we may possibly conclude that the as-prepared nanocrystals presented a multiphase WZ-ZB structure instead of pure hexagonal structure. The else as-synthesized Cu_2_(Sn_1−x_Ge_x_)S_3_ NCs also had been analyzed by the X-ray diffraction (XRD), high-resolution transmission electron microscopy (HRTEM), and select area electron diffraction (SAED). (Figure S 3-8).

**Fig. 3:**
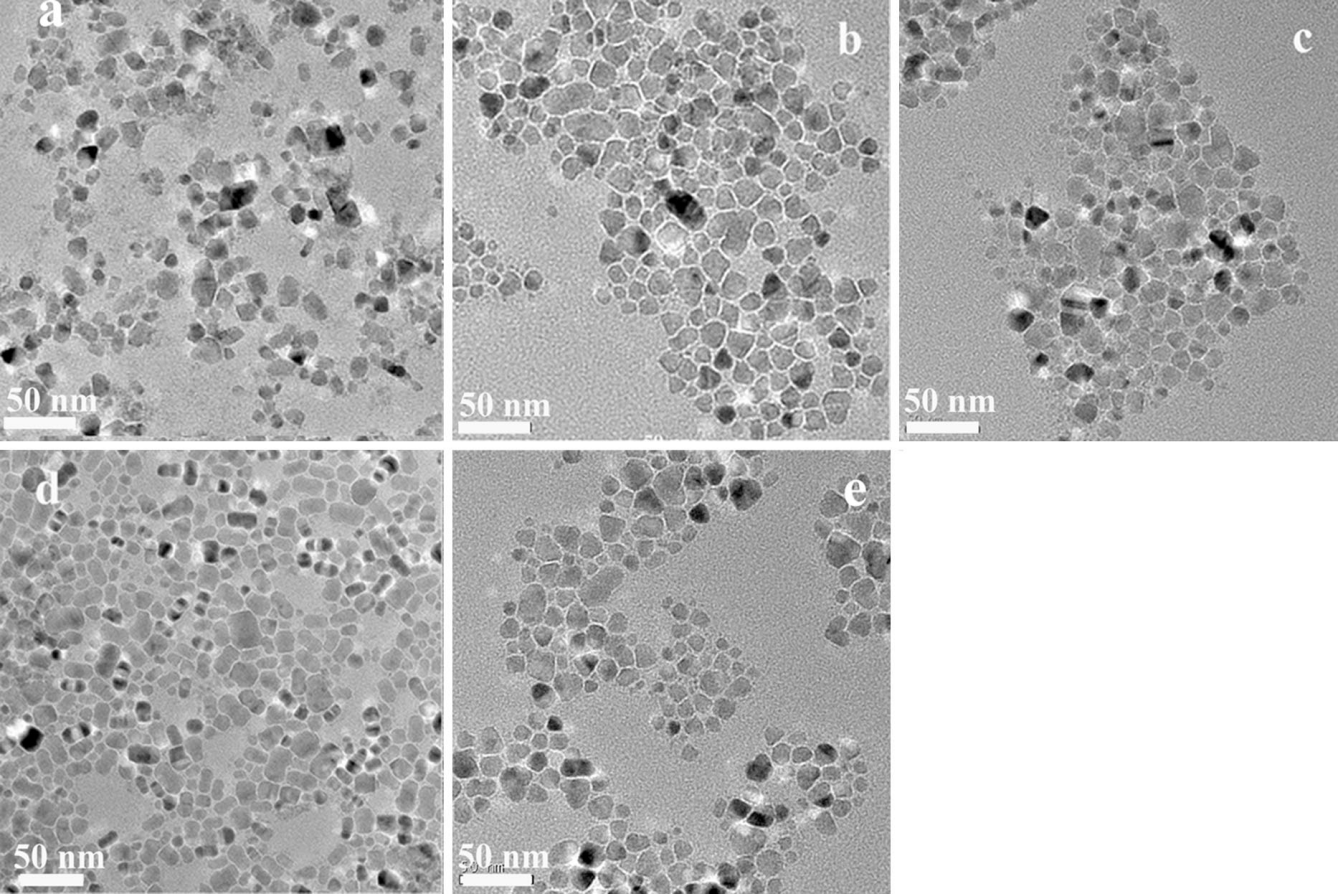
TEM image of Cu_2_(Sn_1−x_Ge_x_)S_3_ nanocrystals with **a** x = 0, **b** x = 0.26, **c** x = 0.5, **d** x = 0.74, **e** x = 1

TEM was performed to further investigate the microstructures of the Cu_2_(Sn_1−x_Ge_x_)S_3_ NCs. TEM images of the as-prepared Cu_2_(Sn_1−x_Ge_x_)S_3_ nanocrystals with x ranging from 0.0 to 1.0 (Fig. 3) show that the as-prepared nanocrystals were monodispersed and quasi-spherical, with an average particle sizes ranging from 12.8 nm to 18.9 nm.

**Fig. 4:**
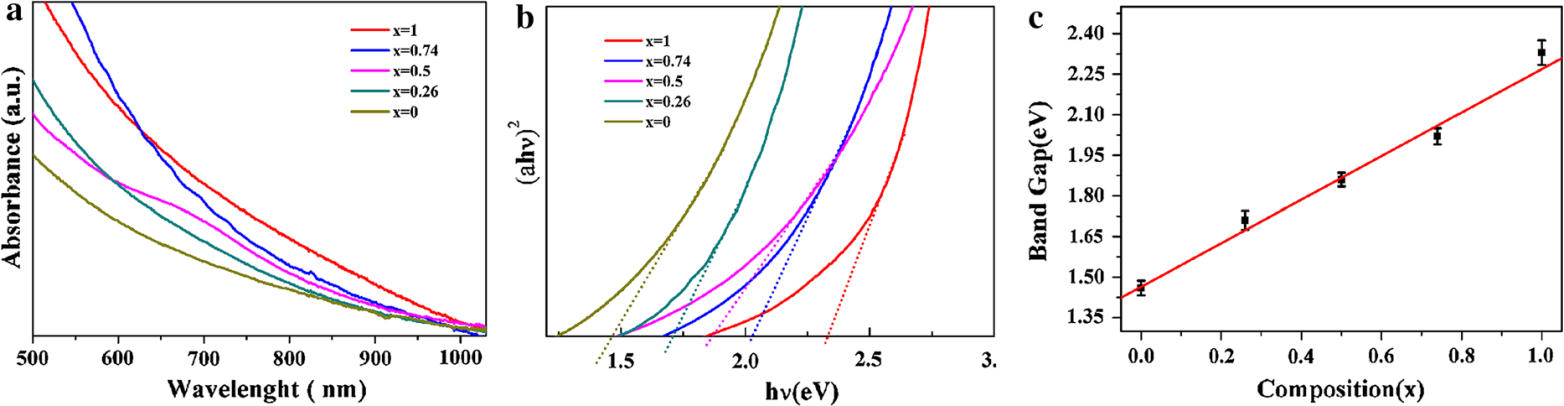
**a** The UV–Vis-NIR absorption spectra of as-prepared Cu_2_(Sn_1−x_Ge_x_)S_3_ nanocrystals; **b** An extrapolation of the spectra to identify the band edge of Cu_2_(Sn_1−x_Ge_x_)S_3_ nanocrystals for various values of x; **c** optical band gap of Cu_2_(Sn_1−x_Ge_x_)S_3_ nanocrystals as a function of x

The UV–vis-NIR absorption spectra of the as-prepared Cu_2_(Sn_1−x_Ge_x_)S_3_ nanocrystals was measured to study their optical properties(Fig. 4a). The band gap energies (*E*_g_) were calculated by plotting the square of the absorption coefficiency (α) as a function of photon energy (hν) and extrapolating the linear portion to intercept the x abscissa (Fig. 4b). The *E*_g_ values determined from the optical absorption of Cu_2_(Sn_1−x_Ge_x_)S_3_ nanocrystals range from 1.46 eV for *x* = 0.0 to 2.33 eV for *x* = 1.0, which covers the optimum bang gap of 1.5 eV for solar photoelectric conversion. It is well known that the crystal symmetry of nanoparticles can affect their optical and electrical properties (Fan et al. 2012; Mei Li et al. 2012; Wang et al. 2012; Maria Ibáñez et al. 2012). However, although the crystalline structure of our as-prepared Cu_2_(Sn_1−x_Ge_x_)S_3_ NCs is the cubic zinc blende (ZB) and hexagonal wurtzite(WZ)mix-phase, the phase composition of these two phase, analyzed by the X’Pert High Score plus, were found almost keep the same values as the *x* varies. Therefore, we consider that the band gap change mainly come from the increasing amounts of Ge incorporation. To further understand the relation between the Ge composition and their band gap evolution, the band gaps of the nanocrystals were plotted as a function of *x* (Fig. 4c). It is found that increasing the Ge contents results in a monotonous increase of the band gap energies, i.e., a nearly linear relationship between the band gaps and the Ge contents in the NCs.

The creation of nanostructured suspension ink for use in a scalable coating process is a key step in the development of low-cost thin-film solar cells (Qijie Guo 2008; Altosaar et al. 2008). These as-prepared Cu_2_(Sn_1−x_Ge_x_)S_3_ nanocrystals can be dispersed in most organic solvents, readily used as the nanocrystal ink for the preparation of photovoltaic absorbers. However, the native olyamine or dodecanethiol ligands, coating on the surface of NCs, have introduced insulating layers around each NC, decreasing the solar cell performance. Even after a high temperature sintering of the film, a large amount of carbon could still remain (Nag et al. 2011). To develop nanocrystals inks that are free from carbon-containing stabilizers, a ligand exchange procedure was performed.

**Fig. 5:**
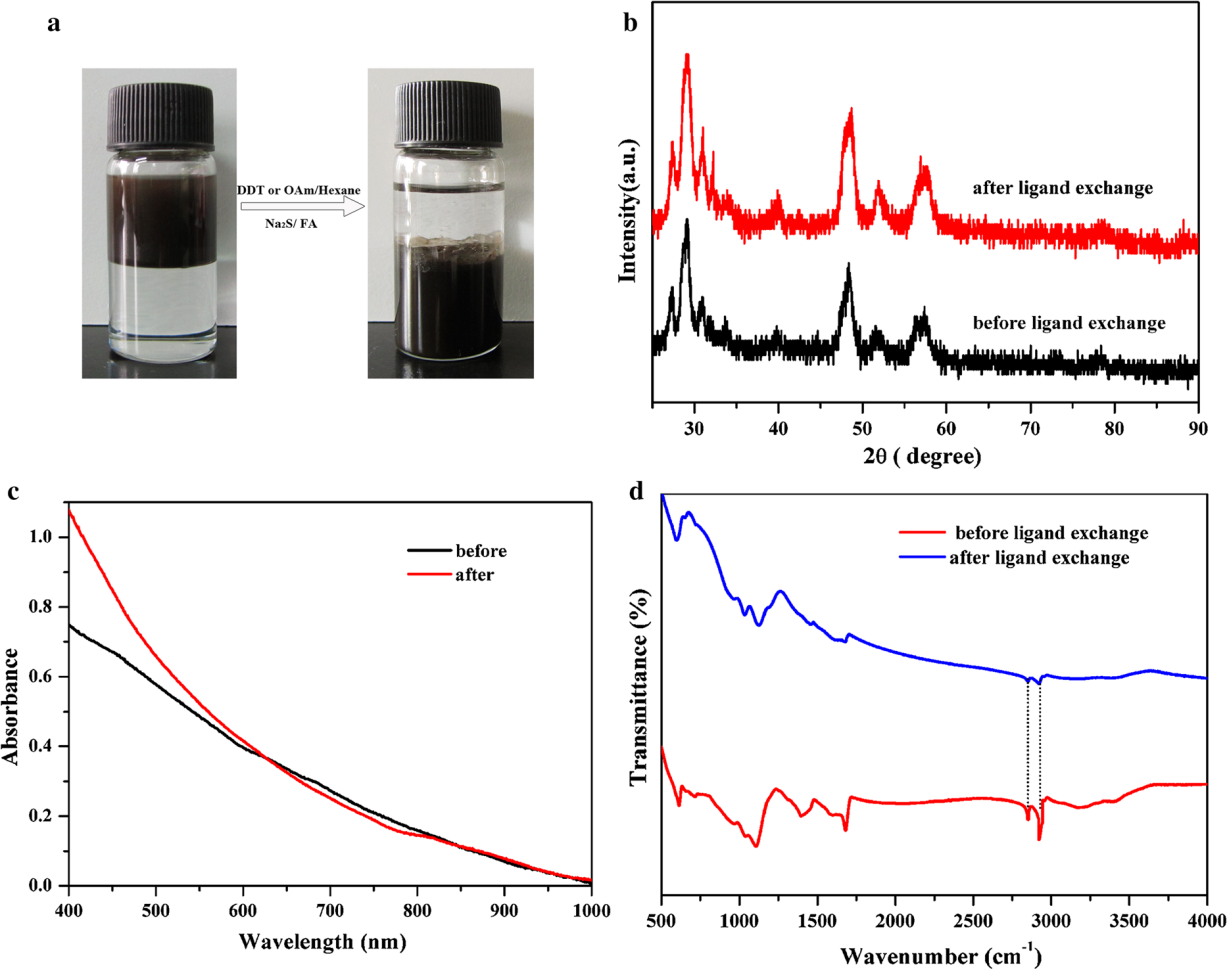
**a** Colloidal dispersion of Cu_2_Sn_0.5_Ge_0.5_S_3_ NCs undergoes phase transfer from hexane to FA by ligand exchange [before (*left*) and after (*right*) ligand exchange]. **b** Powder XRD patterns of Cu_2_Sn_0.5_Ge_0.5_S_3_ NCs before (*black*) and after (*red*) ligand exchange. **c** Optical absorption spectra of Cu_2_Sn_0.5_Ge_0.5_S_3_ NCs before (*black*) and after (*red*) ligand exchange. **d** FTIR spectra of Cu_2_Sn_0.5_Ge_0.5_S_3_ NCs before (*red*) and after *blue* ligand exchange. (Color figure online)

The color change of hexane (black to colorless) and FA (colorless to black) phase (Fig. 5a) facilitates the observation of the ligand exchange reaction. Black colloidal dispersion of Cu_2_(Sn_1−x_Ge_x_)S_3_ nanocrystals undergo a phase transition from hexane to FA with the ligand exchange of the native oleylamine or dodecanethiol ligands with S^2−^. Figure 5b shows the XRD patterns of Cu_2_Sn_0.5_Ge_0.5_S_3_ nanocrystals before and after ligand exchange. There is no observable change between the diffraction peaks, indicating that the ligand exchange does not affect the crystal structure of the NCs. Figure 5c compares the optical absorption of the NCs dispersed in hexane and FA. After ligand exchange, a notably stronger absorption appears in the visible light range from 400 to 600 nm, which possibly resulted from the quantum mechanical coupling energy due to the NCs being more closed. To study the change occurs on the surface of Cu_2_Sn_0.5_Ge_0.5_S_3_ nanocrystals before and after ligand exchange, FTIR and TGA characterizations were performed. As shown in Fig. 5d, the intensity of the peaks at 2852 and 2925 cm^−1^, corresponding to the C-H vibration of olyamine or dodecanethiol, were strongly reduced. In the TGA analyses (Figure S 9), the total weight loss of the S^2−^ capped Cu_2_Sn_0.5_Ge_0.5_S_3_ NCs (8 %) is much lower than that of the organic ligand capped Cu_2_Sn_0.5_Ge_0.5_S_3_ NCs (16 %). These results both indicated that most of the organic ligands were removed from the surface of NCs.

**Fig. 6:**
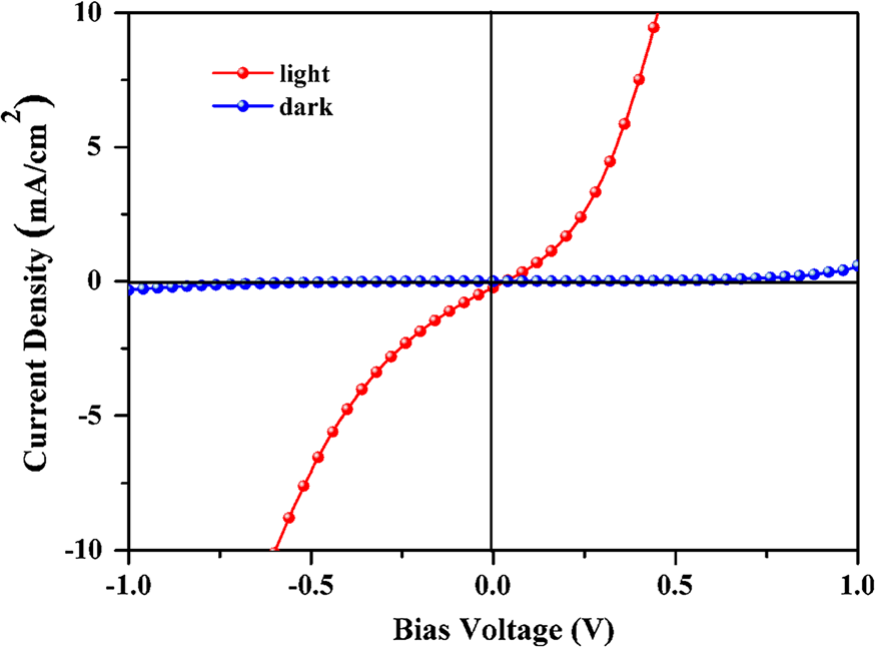
The current–potential (I–V) curve of the Cu_2_ Sn_0.5_Ge_0.5_S_3_ film tested in the dark (*blue*) and under illumination (*red*). (Color figure online)

To validate the optoelectronic properties of as-prepared Cu_2_(Sn_1−x_Ge_x_)S_3_ nanocrystals, the current–voltage (I–V) measurements for the Cu_2_Sn_0.5_Ge_0.5_S_3_ thin films were performed. The film was fabricated on patterned indium tin oxide (ITO)-coated glass substrate via spin casting using a toluene solution of Cu_2_Sn_0.5_Ge_0.5_S_3_ NCs. After that, the aluminum (Al) cathode with 100-nm thick was deposited by thermal evaporation. Figure 6 shows the I–V curves of the Cu_2_Sn_0.5_Ge_0.5_S_3_ film tested in the dark and under an illumination intensity of 100 mW cm^−2^, which was measured in a 1 V bias range. The device exhibits a strong increase in current under light irradiation in comparison to the dark state. Light irradiation excites electrons in the valance band to the conduction band and then increases the holes in the Cu_2_Sn_0.5_Ge_0.5_S_3_. As a result, the current is increased obviously and the conductivity of the film is enhanced. The obvious photoresponsive behavior suggests that the as-synthesized Cu_2_Sn_0.5_Ge_0.5_S_3_ will be a potential candidate in the fabrication of photovoltaic devices.

## Conclusion

In summary, we have shown that compositionally controlled Cu_2_(Sn_1–x_Ge_x_)S_3_ nanocrystals can be successfully synthesized by the hot-injection method through careful tuning the Ge/(Sn+Ge) precursor ratio. The powder X-ray diffraction showed that the resultant NCs could be indexed to either a pure hexagonal structure or the cubic-wurtzite mix-phase. High-resolution transmission electron microscopy (HRTEM) and Raman spectroscopy characterizations further supported the mix-phase structure. The band gaps of the resultant nanocrystals are found to be effectively tailored by tuning their compositions, and the dependence of the band gaps on alloy compositions is almost linear. A ligand exchange procedure was further performed to replace the native ligands on the surface of the NCs with sulfur ions. The photoresponsive behavior indicates the potential use of as-prepared Cu_2_(Sn_1−x_Ge_x_)S_3_ nanocrystals in solar energy conversion systems. The synthesis of compositionally controlled Cu_2_(Sn_1−x_Ge_x_)S_3_ nanocrystals reported herein provides a way for probing the effect of Ge inclusion in the Cu-Sn-S system thin films.

## Electronic supplementary material

Below is the link to the electronic supplementary material.

Supplementary material 1 (DOC 5628 kb)
