# Supplementary material for: Synthesis of compositionally controllable Cu2(Sn1−xGex)S3 nanocrystals with tunable band gaps
Source: J Nanopart Res. 2016 Jun 15;18:161. doi: 10.1007/s11051-016-3439-5 (PMC4909814; doi:10.1007/s11051-016-3439-5)
Supplement: Supplementary file 1 — Supplementary material 1 (DOC 5628 kb) [file 11051_2016_3439_MOESM1_ESM.doc]

**Supporting Information**

**Synthesis of compositionally controllable Cu_2_ (Sn_1-x_Ge_x_) S_3_ nanocrystals with tunable band gaps**

Qingshuang Liang^*^

*^1^* State Key Laboratory of Inorganic Synthesis and Preparative Chemistry, College of Chemistry, Jilin University, Changchun 130012, China.


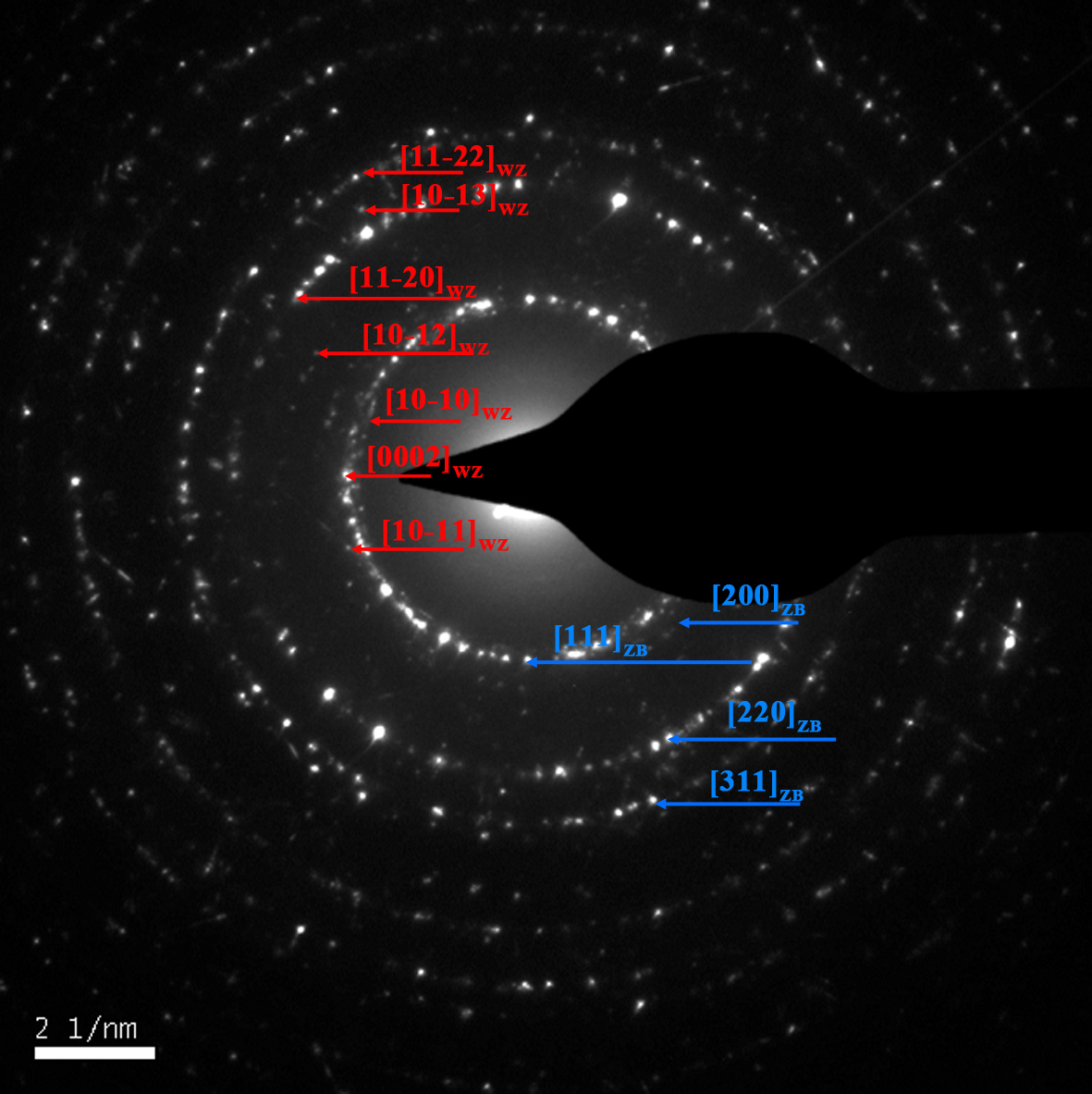


**Figure S1.** SAED pattern of the as-prepared Cu_2_SnS_3_ nonocrystals.


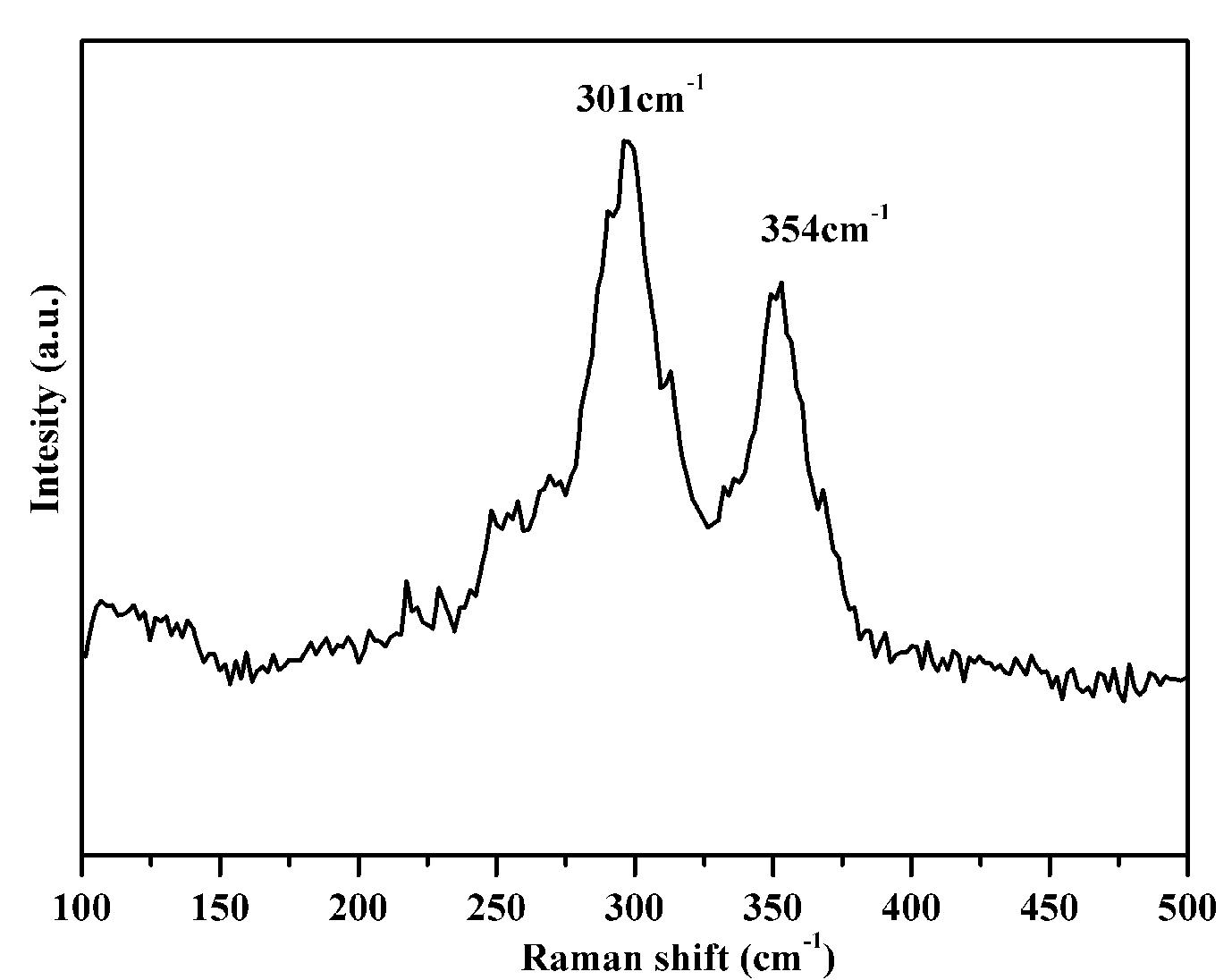


**Figure S2.** The Raman spectra of the as-prepared Cu_2_SnS_3_ nonocrystals.


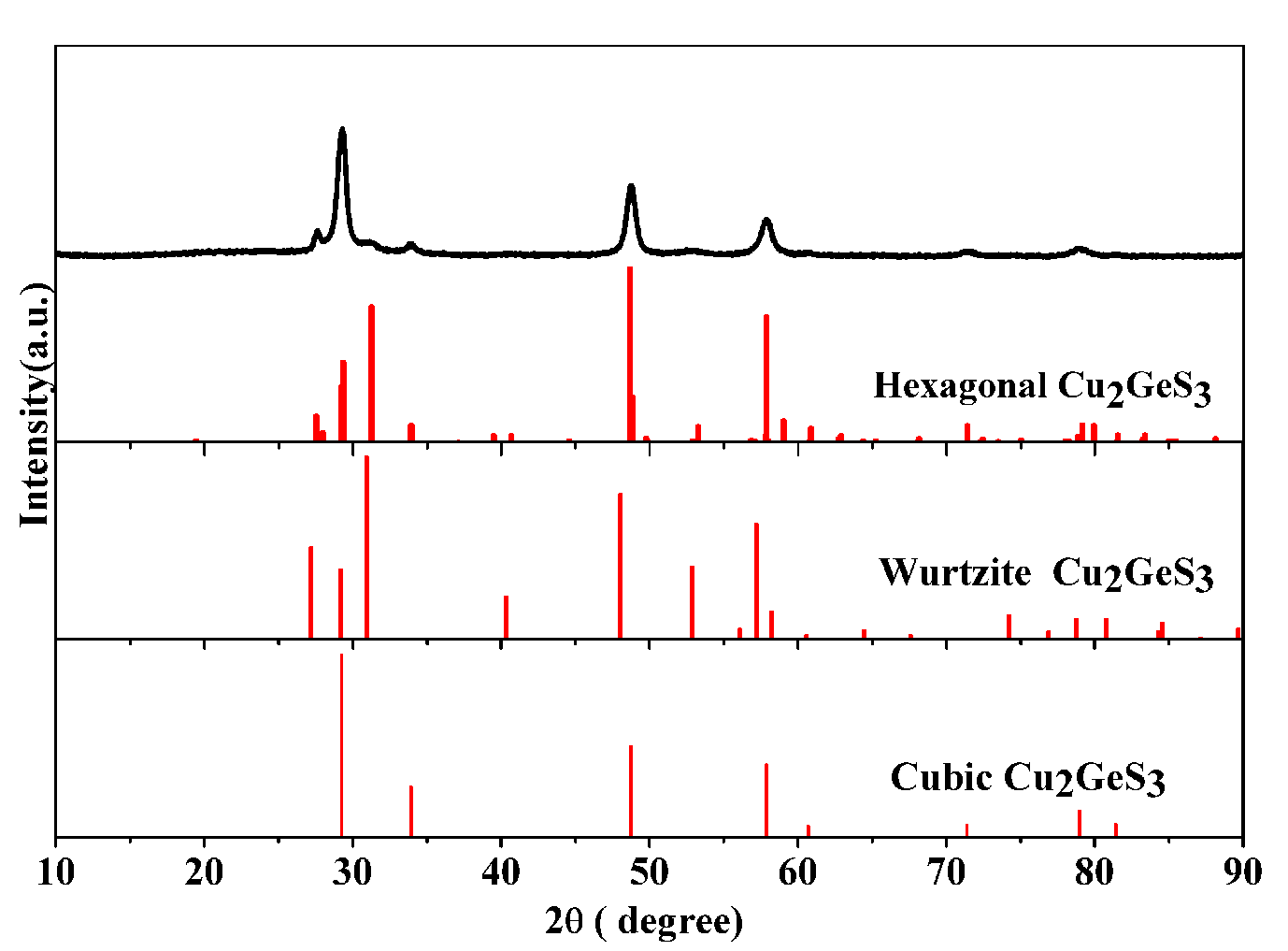


**Figure S3.** XRD pattern of the as-prepared Cu_2_GeS_3_ NCs and simulated XRD pattern structures.


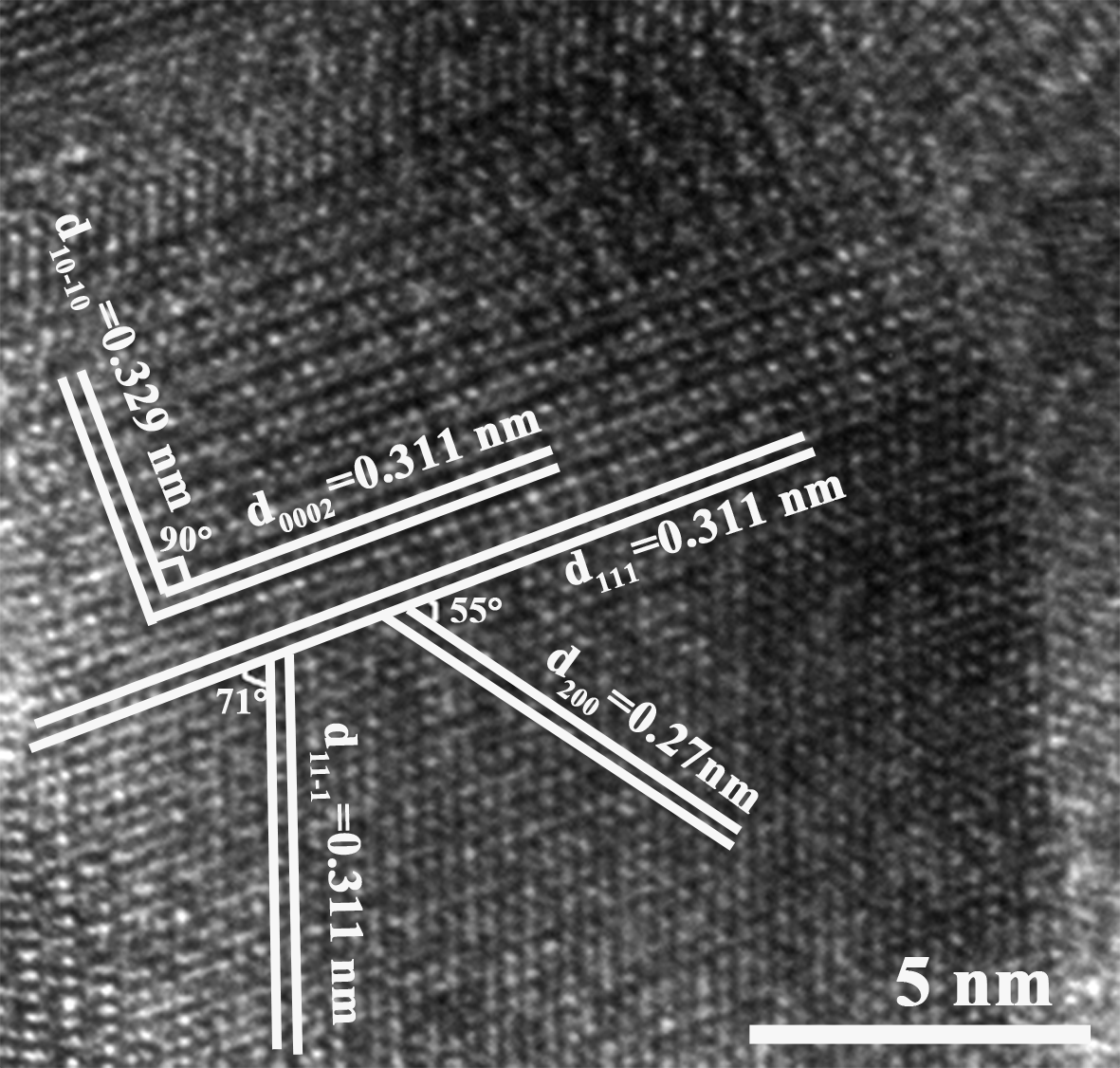


**Figure S4.** HR-TEM image with staking default for Cu_2_Sn_0.74_Ge_0.26_S_3_ nonocrystals.


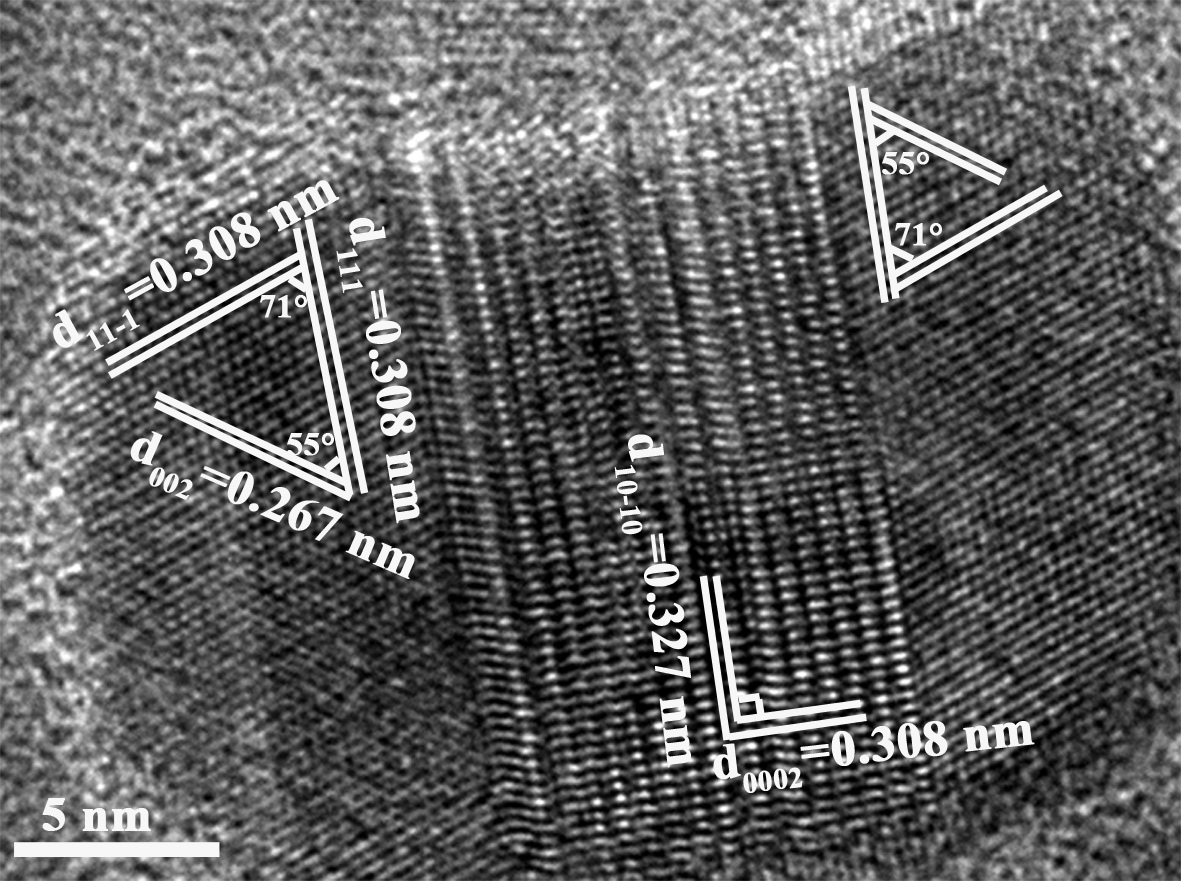


**Figure S5.** HR-TEM image with staking default for Cu_2_Sn_0.5_Ge_0.5_S_3_ nonocrystals.


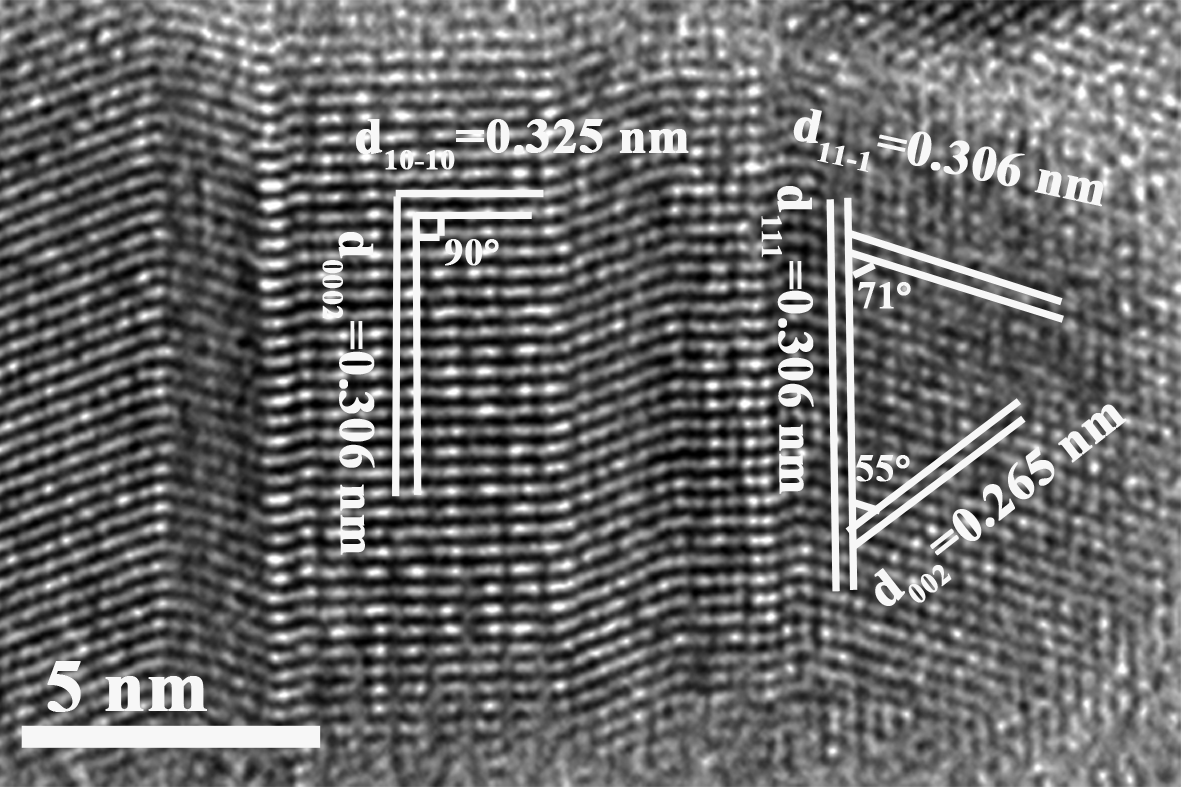


**Figure S6.** HR-TEM image with staking default for Cu_2_Sn_0.26_Ge_0.74_S_3_ nonocrystals.


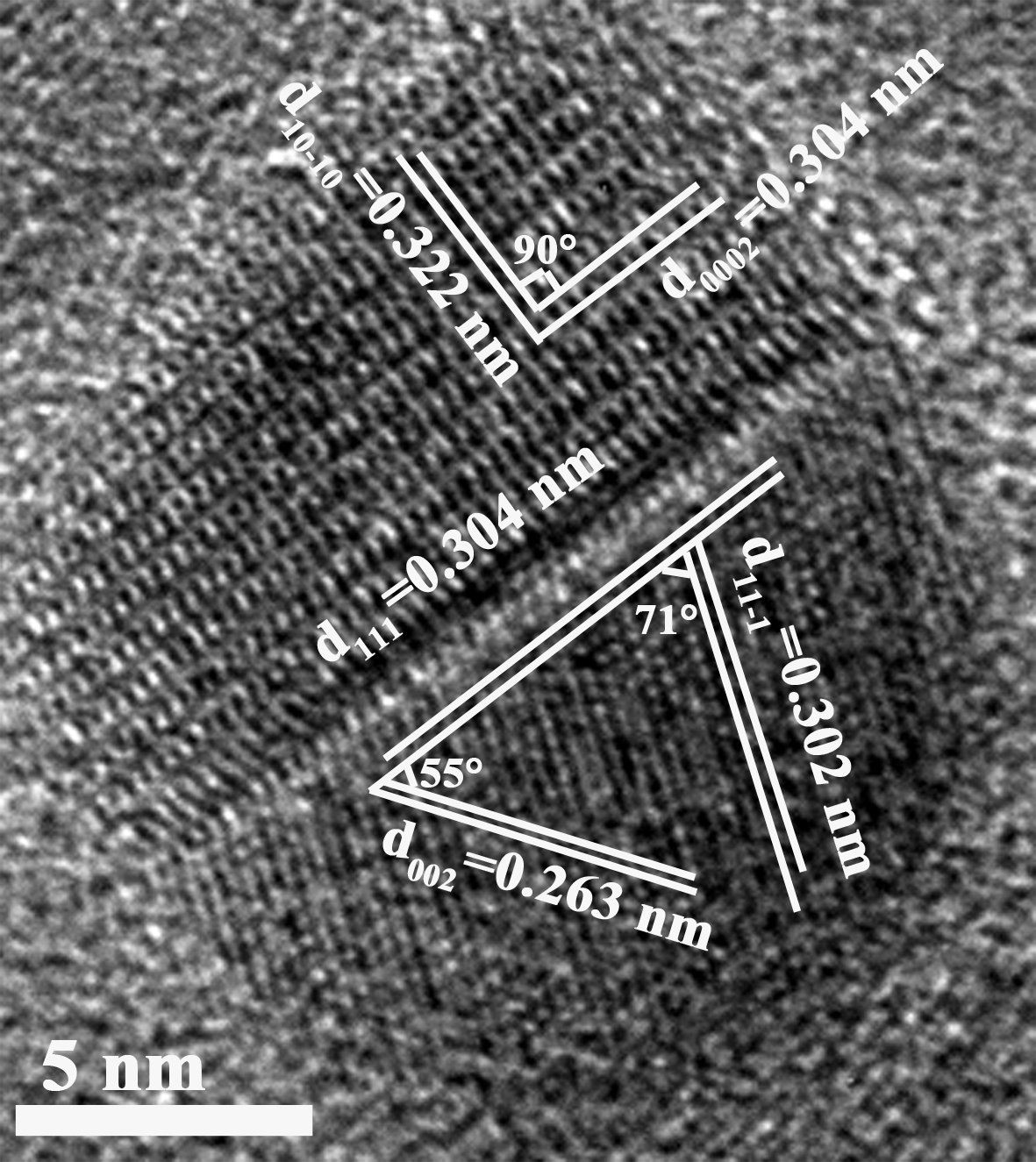


**Figure S7.** HR-TEM image with staking default for Cu_2_GeS_3_ nonocrystals.


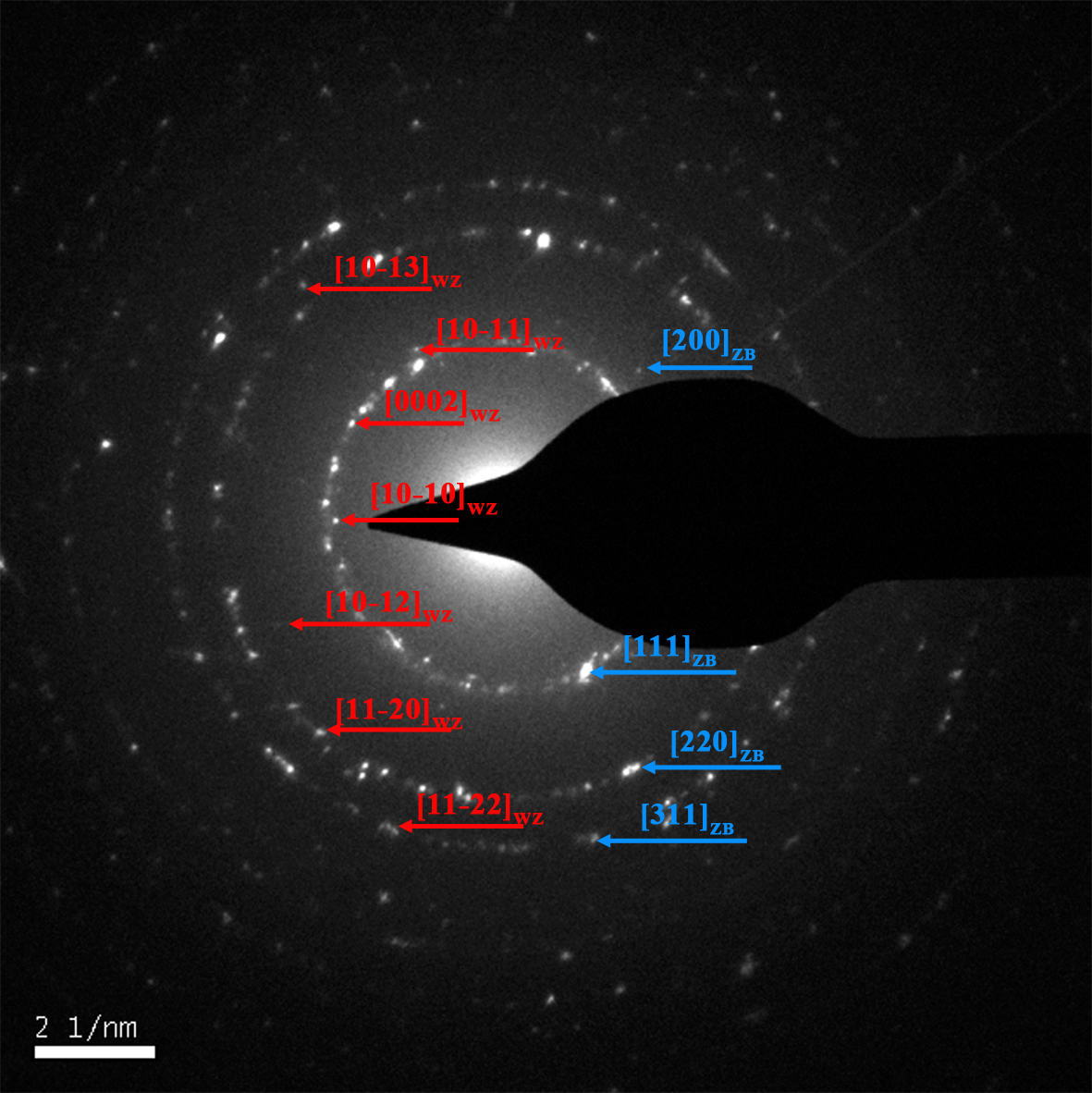


**Figure S8.** SAED pattern of the as-prepared Cu_2_GeS_3_ nonocrystals.


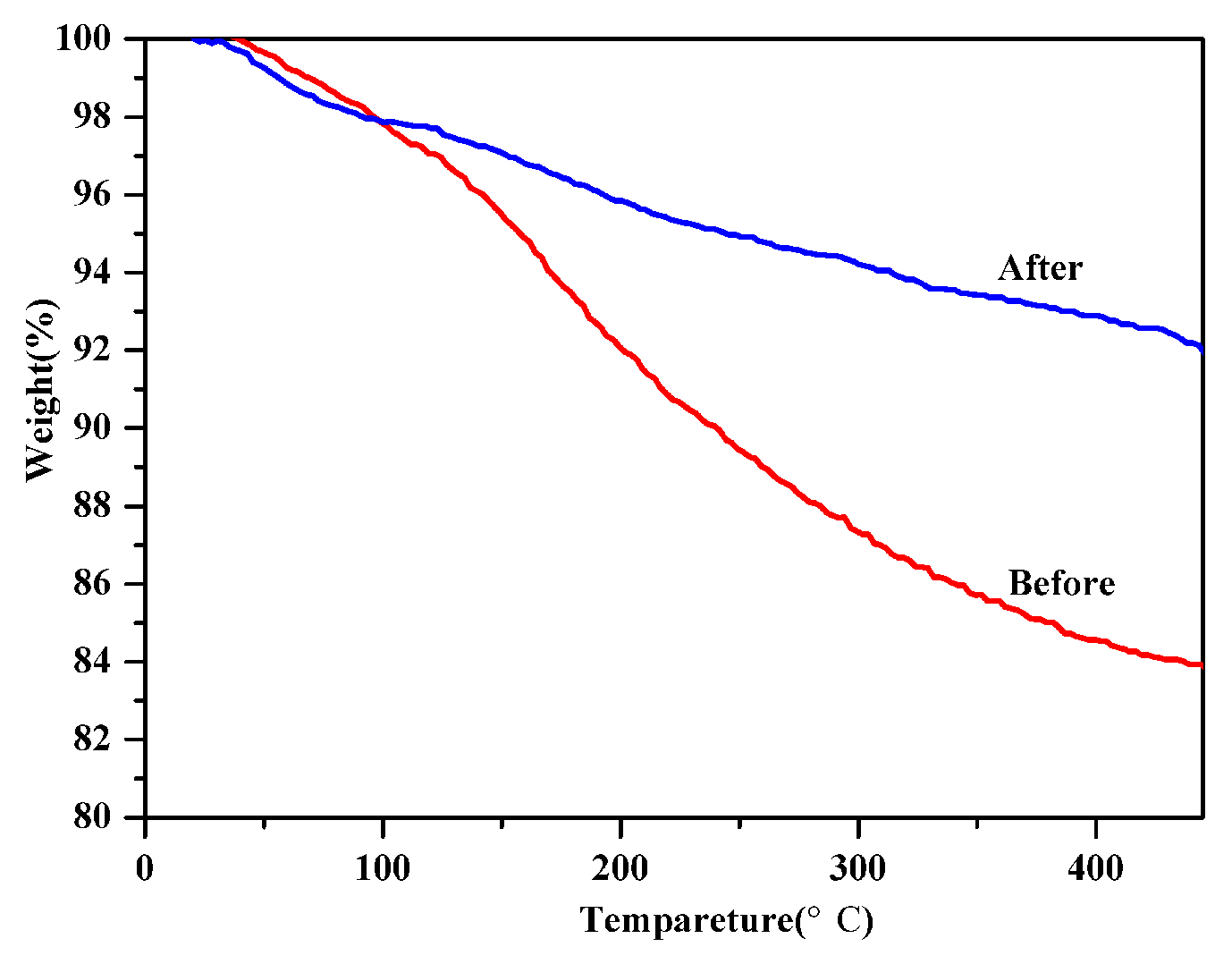


**Figure S9.** TGA analysis of Cu_2_ Sn_0.5_Ge_0.5_ S_3_ NCs before (red) and after (blue) ligand exchange.
